# Comparative analysis of perceptions on artificial intelligence in surgery: a survey study among surgeons and medical students in Ireland

**DOI:** 10.1007/s11845-025-04079-z

**Published:** 2025-09-18

**Authors:** Doris Braunstein, Haniya Farooq, Marco Paolino, Alice Moynihan, Ronan A. Cahill

**Affiliations:** 1 UCD School of Medicine, University College Dublin, Dublin, Ireland; 2 UCD Centre for Precision Surgery, University College Dublin, 47 Eccles Street, Dublin, Ireland

**Keywords:** Artificial intelligence, Intraoperative decision-making, Medical students, Surgeon perspectives, Surgery

## Abstract

**Background:**

Artificial Intelligence (AI) promises to revolutionize healthcare but has been previously characterized by cycles of “boom” and “bust.” Alongside technological capability, realistic user expectations are essential for appropriate implementation. We surveyed surgeons, surgical trainees, and medical students in Ireland regarding their current perceptions.

**Methods:**

Electronic survey distributed through professional networks and social media with institutional ethical approval. Statistical and thematic analyses were performed to identify key perspectives.

**Results:**

Among 94 participants (63% medical students, 18% surgical trainees, 15% consultants, and 4% ancillary surgical roles), 62.7% “strongly agreed” that AI could enhance real-time decision-making during surgery. Most (90.5%) believed AI was already being surgically deployed to some extent although only 18% felt it appropriate ever to use for decision-making. While 53.2% were positive about AI’s potential to improve surgical outcomes, 72.3% reported no AI training in this context despite 86.2% expressing interest. The primary concerns with AI regarded accuracy and reliability (38.7%) and the lack of evidence of effectiveness (33.7%). Surgical trainees expressed greater concern about AI transparency (47% “extremely concerned”) compared to consultants (42.9% “slightly concerned”) and, along with students, declared higher concern regarding liability issues versus consultants (64.3% of whom had “little to no concern”).

**Conclusion:**

Students and postgraduates in surgery in Ireland express optimism and high expectations for AI’s potential to improve surgery. However, concerns about reliability, evidence, and liability persist with clear caution regarding automated decision-making and insight regarding the need for education that may help align expectations realistically regarding AI evolution.

**Supplementary Information:**

The online version contains supplementary material available at 10.1007/s11845-025-04079-z.

## Introduction

The surge in advancements in Artificial Intelligence (AI) is increasingly evident in society and indeed in healthcare, with the FDA having approved 950 software as medical devices that deploy AI and Machine Learning [1]. Within the realm of surgery, there is a growing interest in harnessing AI’s capabilities for pre-operative risk assessments [2, 3], radiology image-based navigation [4–6] and video-based surgical technique evaluations [7, 8]. However, explorations into AI’s potential for real-time automated decisioning during surgery are more early stage. Alongside technological development, receptivity and perceptions of current and future surgeons for the integration of such tools into surgical practices need consideration. This is especially important given the previous “boom” and “bust” cycling seen with AI since its inception in the 1950 s as misplaced expectations undermine adoption and implementation.

There have been few studies which have explored perceptions towards AI in surgical contexts, including the views of medical students in Germany [9], vascular surgeons in Canada [10], and acute surgeons across a variety of countries [11]. A discernible gap persists in the context of consultant surgeons, surgeons in training, and medical students within the same jurisdiction. This study explores the perspectives of these groups in Ireland regarding AI’s role, especially in real-time surgical decision-making. The findings are considered in the context of other reports in this area.

## Methods

This study received ethical approval as a low-risk research project (Ref: UTMREC-SM-E-23-354) from the University Teaching Medical Research Ethics Committee at University College Dublin School of Medicine, with exemption from full ethical review. Following this approval, an electronic survey using a structured web-based questionnaire via Google Forms was distributed to participating volunteer medical students and surgeons across Ireland. Invitations to participate were circulated through our established academic and professional networks, as well as through social media platforms including Twitter. The survey (see Supplementary Material) employed a Likert scale format, designed to elicit detailed responses concerning various aspects of AI in surgical contexts specifically. The following key domains had been identified by brainstorming among the authorship group: (1) the anticipated potential of AI in surgery, (2) training and exposure experiences, (3) concerns regarding AI, (4) liability issues, and (5) ethical implications. Statistical analysis between groups was performed using chi-square tests of independence to compare response distributions across different participant categories (medical students, surgical trainees, and consultant surgeons). All statistical analyses were conducted using Microsoft Excel (Microsoft Corporation, Redmond, WA, USA) and SPSS Version 27.0 (IBM, Armonk, NY, USA), with *p* < 0.05 being taken to indicate statistical significance. Qualitative analysis was performed on responses to the one open-ended question. Three researchers independently reviewed these responses to extract major themes. The identified themes were then discussed collectively to reach consensus on the final set of key themes emerging from the data.

## Results

**Fig. 1:**
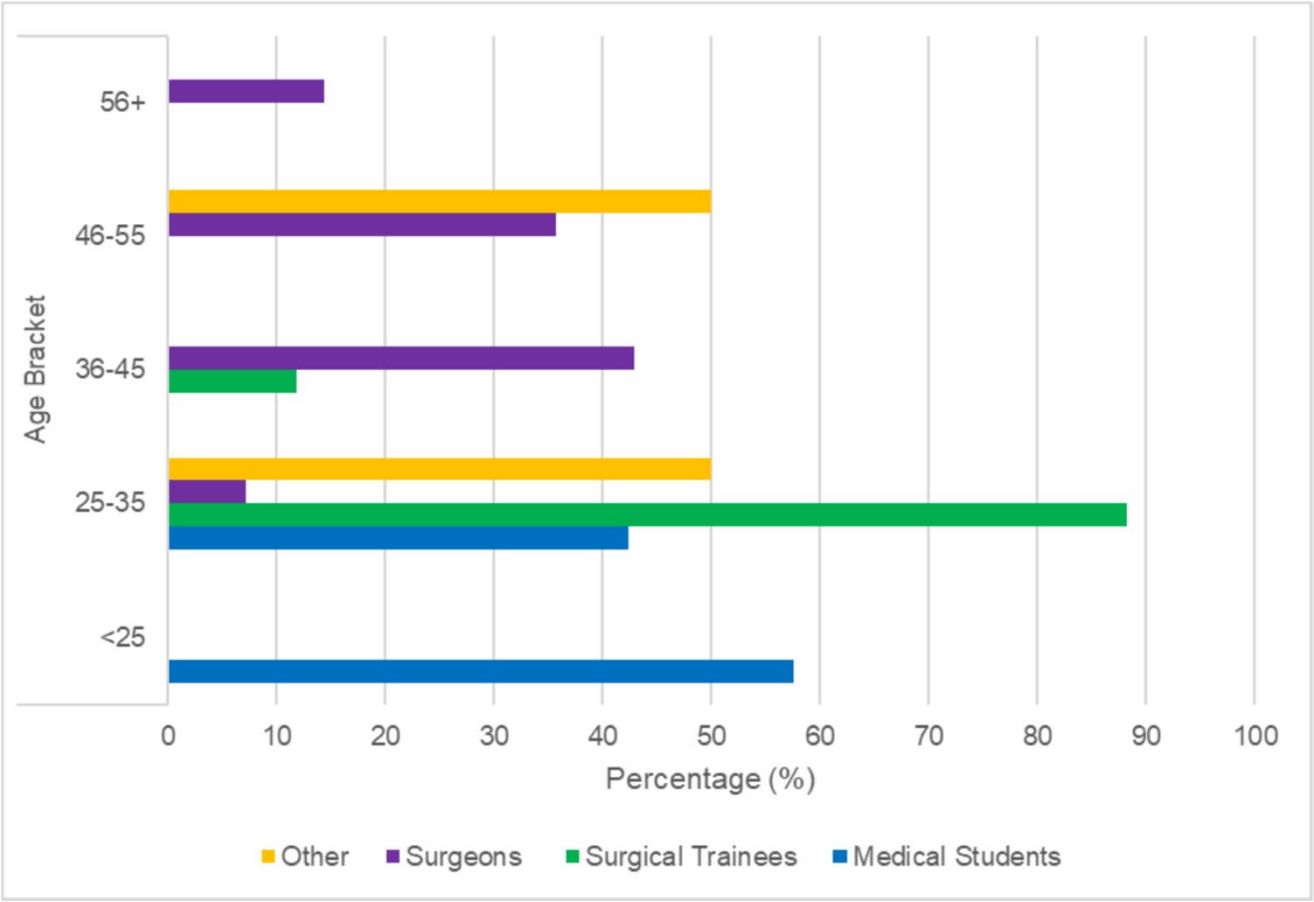
Distribution of participants by age

Ninety-four participants, comprising 59 medical students (63%), 17 surgical trainees (18%), 14 consultant surgeons (15%), and four individuals (4%) in ancillary surgical roles, completed the survey. The four respondents with ancillary surgical roles included a surgical researcher, surgical tutor, surgical industry representative, and a surgical radiology expert. 45.7% of participants were aged between 25 and 35 years, 36.2% were under 25 years, and 18.1% were over 35 years (see Fig. 1). Those under 25 were primarily medical students with those 25–35 years including both surgical trainees and early-career surgeons, while those older than 36 years were mostly experienced surgeons. 42.9% had 6–10 years of clinical experience, with 25.7% having 11–20 years, 17.1% 1–5 years, and 14.3% over 21 years of clinical experience. In terms of surgical specialties, our respondents included eighteen in general surgery, three in plastic surgery, two in vascular surgery, two in urology, and one in cardiothoracic surgery.

### Perceptions on AI’s potential in surgery

**Fig. 2:**
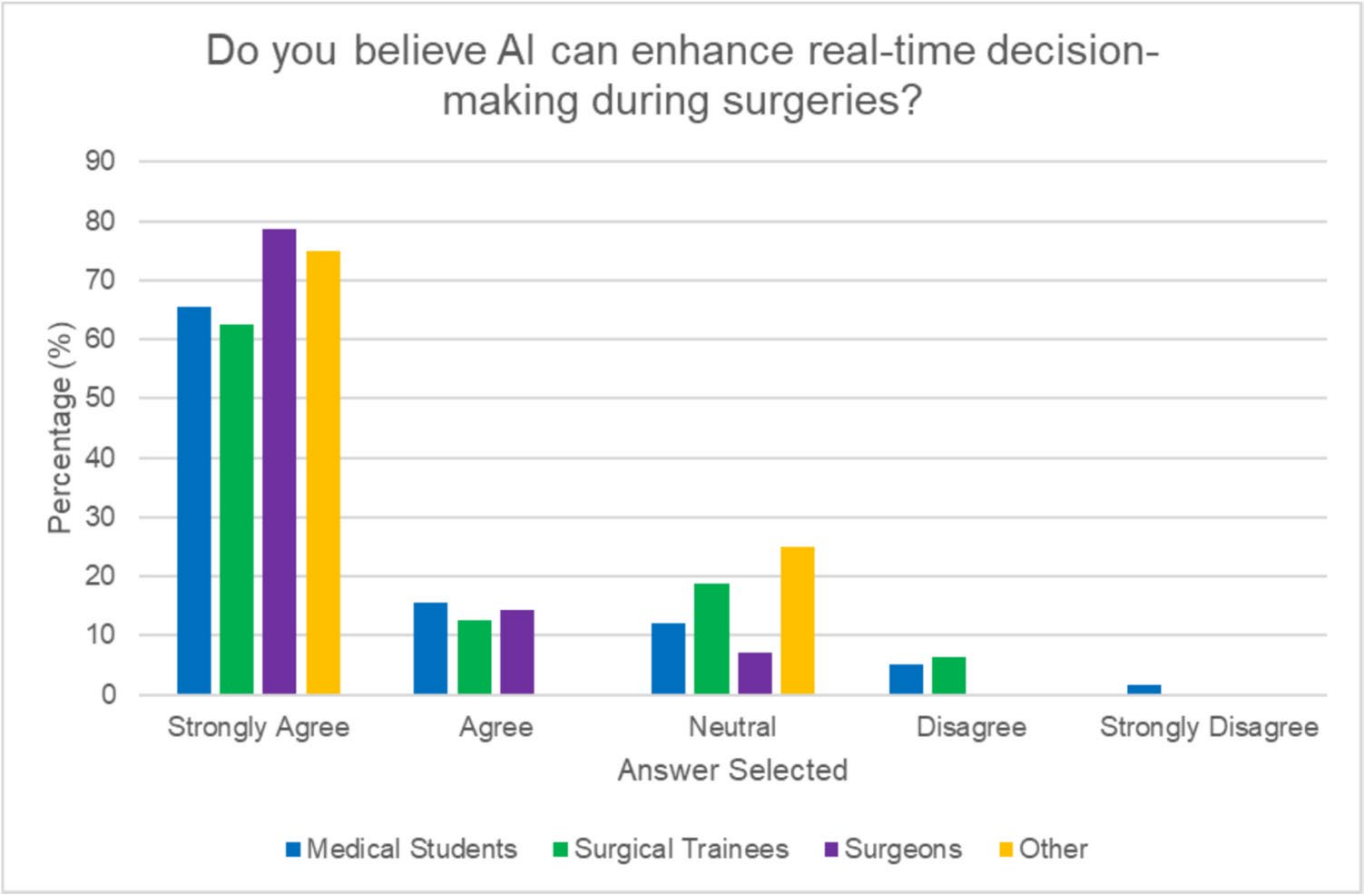
Perceptions of AI’s impact on real-time decision-making in surgery by role

**Fig. 3:**
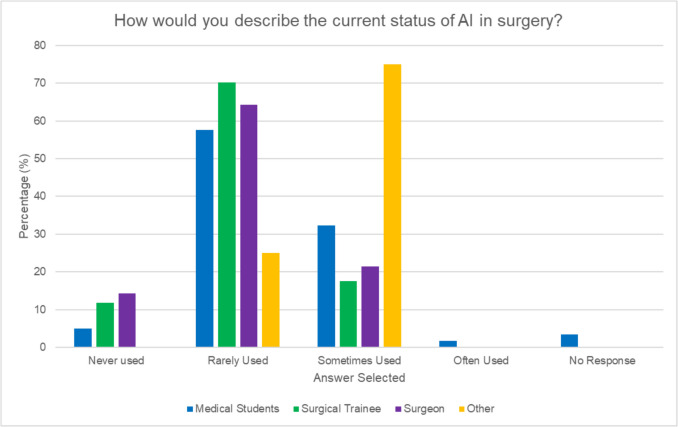
Perceptions on the current status of AI in surgery by role

Over 25% of surgeons and trainees indicated significantly greater familiarity with AI compared to medical students (*p* < 0.00001). Most participants (62.7%) “strongly agree” that AI has the potential to enhance real-time decision-making during surgery (see Fig. 2) and indeed a majority (90.5%) believe AI has already been used in surgery either “sometimes” (29.8%) “rarely” (59.6%) “often” (1.1%)(see Fig. 3) with 85.7% of consultants, 88.2% of trainees, and 91.5% of medical students selecting options describing AI as currently being used in some capacity in surgery although only medical students selected “often used.” Of the 7.4% that selected “never used” for the current status of AI in surgery, there was an equal distribution between groups. The four respondents in ancillary surgical roles all expressed favorable views towards the use of AI in surgery. Trust in AI for real-time guidance was ranked as moderate across all respondent groups correlating with similar positivity (53.2%) towards AI’s potential to improve surgical outcomes with no significant difference in level between the groups (*p* = 0.53). 39.1% believed that utilizing AI-driven simulations to provide realistic and diverse surgical scenarios would be most beneficial to enhance technical skills, decision-making capabilities, and overall readiness for real-life surgeries. Respondents indicated preoperative planning and simulation (34.4%) as another area likely to benefit from AI while others (23.7%) believed that AI could improve the accuracy and functionality of surgical instruments. 19.6% highlighted the value of implementing AI systems for real-time feedback and detailed post-performance analysis while 18.3% agreed that real-time decision-making during surgery is a critical area where AI could be effectively applied.

### Training and exposure

**Fig. 4:**
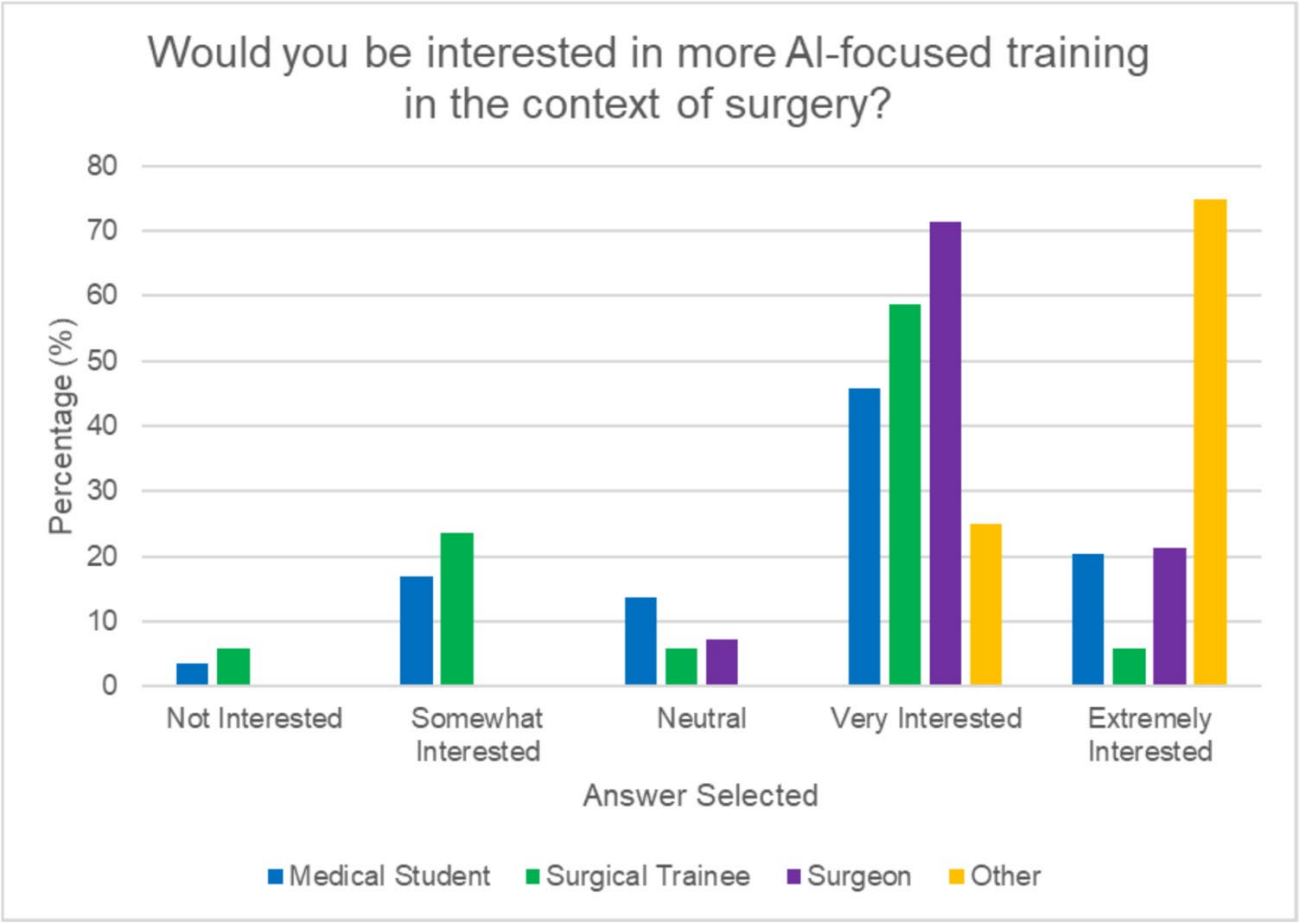
Participant interest in AI-focused training by role

**Fig. 5:**
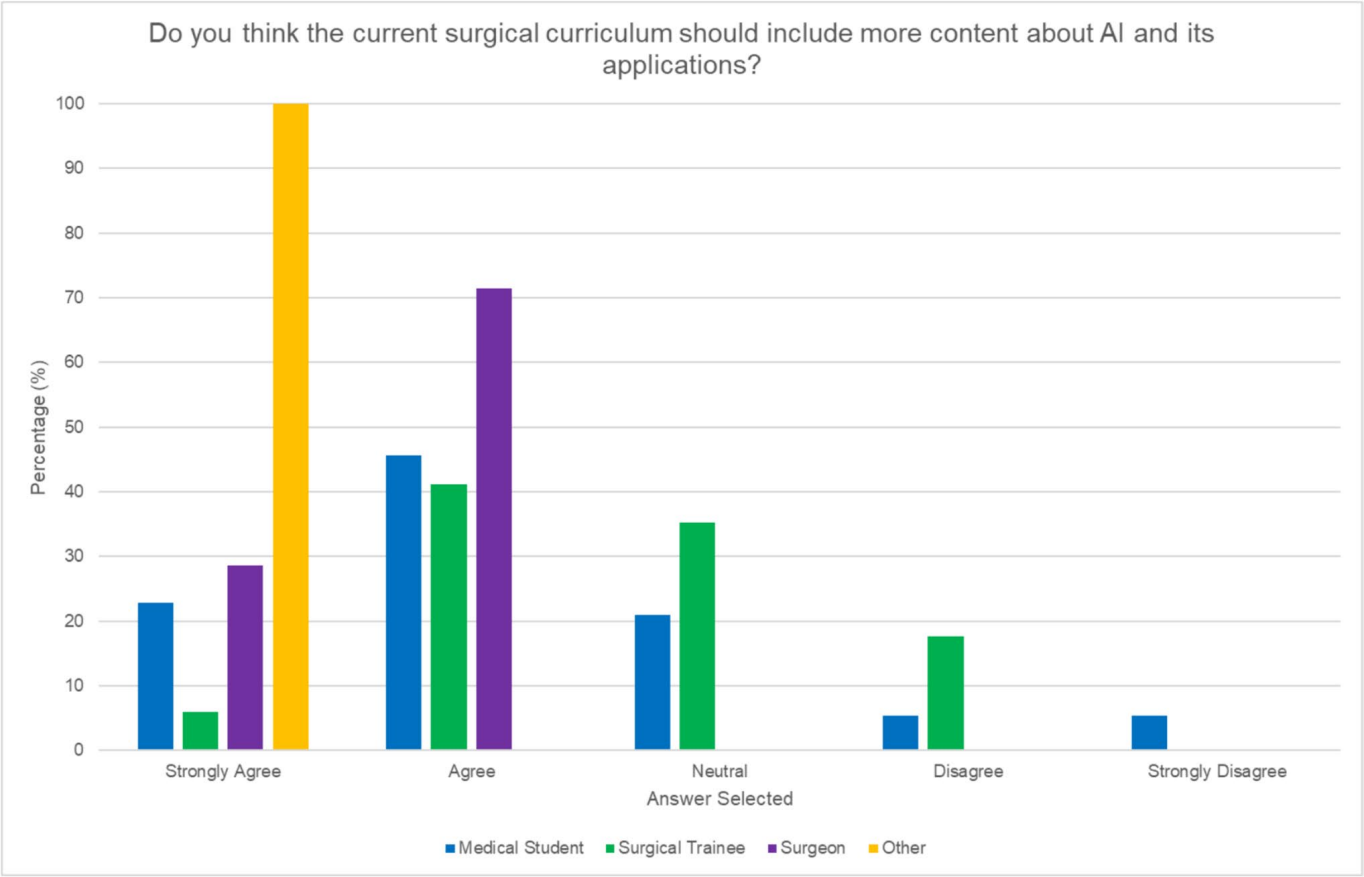
Perceptions on AI within the surgical curriculum by role

The majority of respondents (72.3%) reported having no training or exposure related to AI in surgical contexts while a high majority overall (86.2%) expressed interest in undertaking this. Notably, consultants displayed a more favorable approach to increased AI training compared to surgical trainees (see Fig. 4) with consultants mainly selecting “extremely interested” (21.4%) or “very interested” (71.4%). Fewer surgical trainees selected “extremely interested” (5.8%) with 58.8% selecting “very interested” and 23.5% selecting “somewhat interested” with 5.8% being “not interested.” All consultants agreed that the current surgical curriculum should include more content about AI and its applications although only 47% of surgical trainees did so (see Fig. 5).

### Concerns

**Fig. 6:**
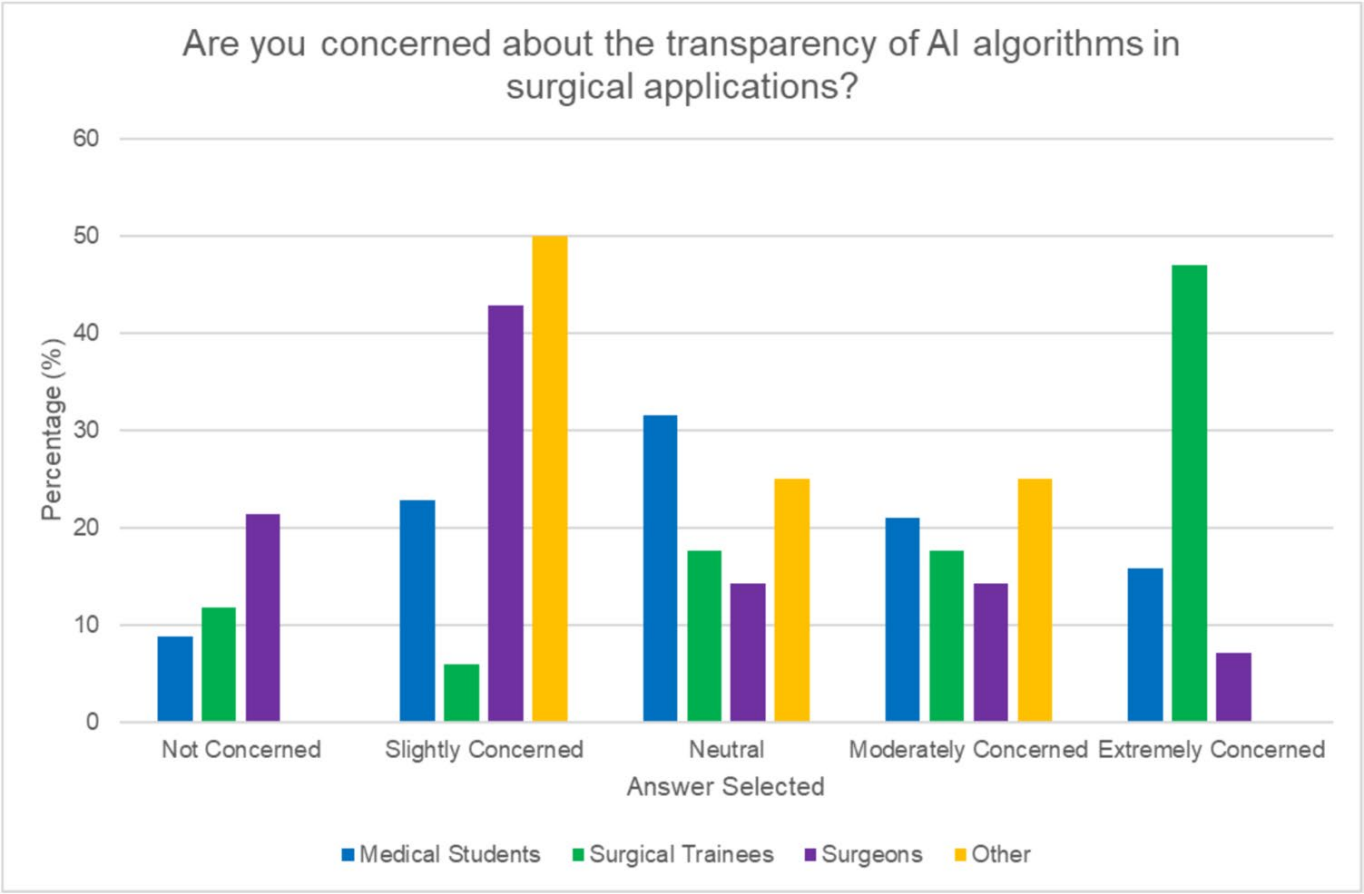
Concerns regarding transparency of AI algorithms in surgical applications by role

**Fig. 7:**
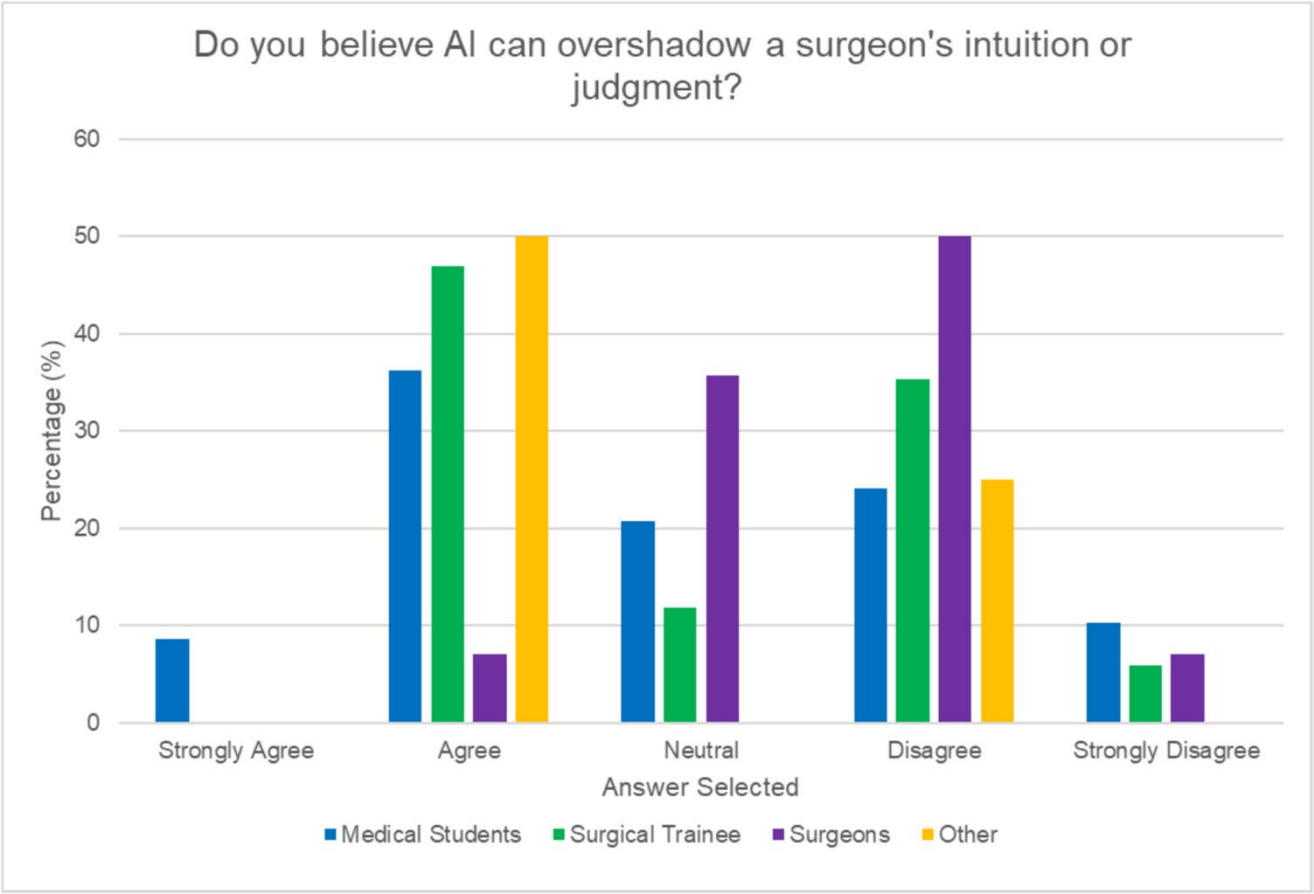
Perceptions on AI overshadowing a surgeon’s judgment by role

38.7% participants identified the accuracy and reliability of AI predictions as the biggest risk when integrating AI into surgical procedures. When asked about barriers to integration, the most selected options included “lack of evidence of effectiveness” (33.7%) “fear of malpractice” (28.3%) and “lack of training” (22.8%). Surgical trainees were most concerned with the lack of evidence of effectiveness (53.9%) while surgeons selected lack of evidence (27.3%) and lack of training (27.3%) as the top two barriers. Interestingly, medical students were most concerned with fear of malpractice (35.2%). Overall, 26.9% of participants were concerned about the transparency of AI algorithms, with 26.9% of respondents remaining neutral, 23.7% being slightly concerned, and 19.4% extremely concerned. Surgical trainees (“extremely concerned” 47%) expressed greater concern than surgeons (“slightly concerned” 42.9%) (see Fig. 6). Opinions were divided overall; however, on whether AI can overshadow a surgeon’s intuition or judgment, with 34.4% agreeing and 30.1% disagreeing. The majority of consultants (58%) disagreed with this statement, while 47% of surgical trainees and 44.8% of medical students agreed with it (see Fig. 7). 18.3% of participants (14.3% of surgeons, 11.8% of trainees, and 15.2% of medical students) expressed worries about surgeons becoming overly dependent on AI over time.

### Liability and ethics

**Fig. 8:**
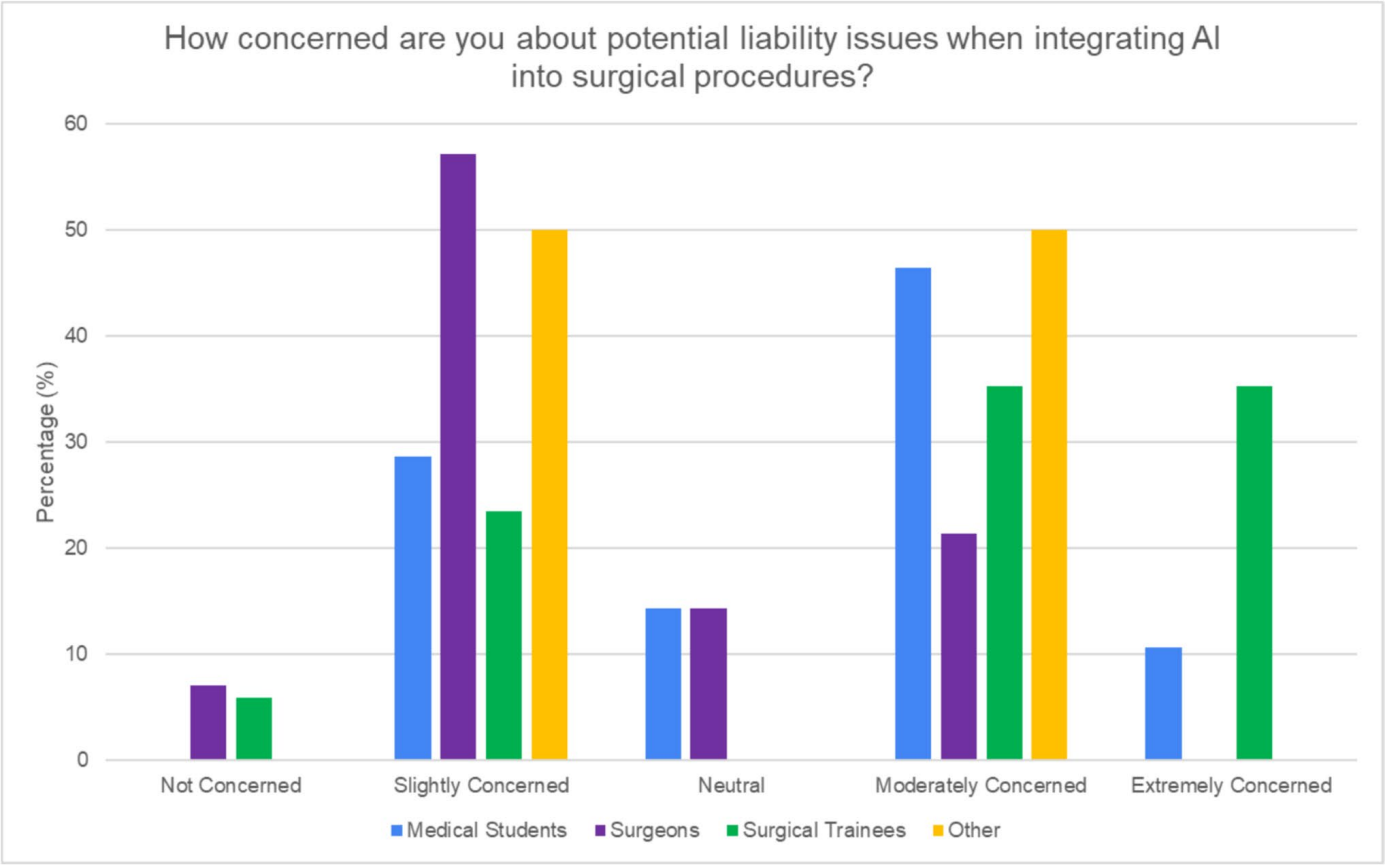
Concerns regarding potential liability issues in the surgical integration of AI by role

40.2% of respondents expressed moderate concern regarding liability issues related to AI. Surgical trainees were more concerned about this than consultants. The majority of surgical trainees (71.1%) and medical students (57.2%) described “extreme to moderate concern” regarding liability (see Fig. 8). In contrast, a majority of consultants (64.3%) selected “none to slight concern.” When asked who should bear the primary responsibility if complications arise from AI integration in surgical procedures, the majority in each cohort indicated that it should be shared among multiple parties.

### Open-ended feedback

This section of the survey received 15 responses, which were categorized into several themes. These themes include regulatory and educational needs, data quality, implementation challenges, clinical applications, and general perceptions (see Table 1).

**Table 1:** Themes emerging from thematic analysis of open-ended responses on AI integration in surgery

Category	Themes from responses
Regulatory and education	- AI devices should undergo comprehensive regulatory review before market placement - Doctors need education on AI to understand its limits, biases, and flaws - Medical students should have more future-focused education
Data and validation	- AI requires excellent datasets for training and validation - Clinical evidence must support efficacy and safety - Concerns about data robustness and potential mortality linked to AI - Not enough datasets currently to make technology robust for surgery
Implementation concerns	- Current AI will continue to evolve and improve - Concerns about AI reducing clinician competence over time - Cybersecurity and validation concerns as AI advances
Clinical applications	- AI has huge potential in surgery as a decision support tool - Needs cooperation between clinicians and industry to improve technology access and utilization
General perceptions	- AI is an exciting area of innovation that can increase efficiency - Implementation should occur in a population that is receptive and has educated surgeons and trainees - Inevitable integration of AI into surgical practice

## Discussion

This study provides novel insights into the perceptions of AI use in surgery among medical students, surgical trainees, and consultant surgeons in Ireland, revealing a promising landscape characterized by cautious optimism and experience-based differences in outlook. A key finding of our study is the widespread belief among respondents that AI is already in use in surgical settings, despite its limited actual implementation. This overestimation aligns with observations in other papers. Farid et al. reported that over 66% of plastic surgeons and trainees believed AI could improve surgical planning and accuracy, despite 82% admitting little to no experience with AI in their specialty [12]. Similarly, Pinto dos Santos et al. noted that the majority of medical students in Germany held optimistic views about AI’s ability to revolutionize radiology, potentially overestimating its current capabilities [9].

This overestimation trend extends beyond surgery. Sit et al. found that UK medical students were similarly optimistic about the capabilities of AI in radiology, despite limited exposure to AI in clinical settings [13]. This perception gap suggests a broader trend of overestimating AI’s current role in healthcare, possibly fuelled by media coverage and the increasing presence of AI in daily life. The discrepancy between perceived and actual AI implementation underscores the need for clearer communication about realistic current capabilities in surgical settings.

Our study also revealed significant differences in AI perception based on professional experience. Established surgeons generally displayed a more favorable to neutral view of AI, while medical students showed more extreme responses, both positive and negative. This differs from what Oh et al. observed, that while physicians in Korea showed higher familiarity with AI, medical students rated AI’s capabilities more optimistically [14]. The more cautious approach of experienced surgeons may reflect a better understanding of the complexities involved in surgical practice and the potential challenges of AI integration. Our findings align with Hashimoto et al., who noted that experienced surgeons often have a more nuanced view of AI’s potential in surgery [15]. This suggests that clinical experience plays a crucial role in shaping realistic expectations of AI in surgical practice. Conversely, the polarized views among medical students could stem from limited clinical experience combined with exposure to both enthusiastic and cautionary narratives about AI in medicine.

The disparity in AI perception across experience levels highlights the need for targeted educational interventions. While most respondents reported no formal AI training, there was high interest in such opportunities across all groups. This interest aligns with findings from Li et al., where 66% of Canadian vascular surgeons expressed eagerness to learn more about AI [10]. Similarly, De Simone et al. discovered that a significant portion (74.5%) of trauma surgeons worldwide anticipate AI becoming available in their settings in the future, with nearly half (49.5%) showing high interest in AI-related courses or research projects [11]. Wartman and Combs argue that medical education must evolve from the information age to the age of artificial intelligence [16]. Our findings support this view, highlighting the interest in AI-focused curricula in surgical training programs.

Perhaps surprisingly, our study found that established surgeons showed a more favorable approach to increased AI training compared to surgical trainees. This seems to contrast somewhat with the general assumption that younger professionals are more open to new technologies. This disparity may stem from several factors:

1. Generational differences in comfort with new technologies, with established surgeons recognizing a greater need for structured education in AI.
2. Surgical trainees potentially feeling more innately confident with AI technologies, possibly due to greater exposure to digital technologies throughout their education and personal lives.
3. The current demanding schedules of surgical trainees, making additional AI training appear burdensome at their career stage.

These findings emphasize the need for tailored AI education programs that address the specific needs and constraints of different career stages in surgery. Interestingly, the optimism and high interest from the respondents in ancillary surgical roles may perhaps indicate their role in AI uptake, particularly as enablers of integration which influence institutional readiness.

Concerns about AI’s accuracy, reliability, and liability implications were prevalent across all groups. However, our study uniquely found that medical students were more concerned about malpractice implications, while surgeons and trainees focused on the lack of evidence for AI’s effectiveness. The malpractice concern shared by medical students may stem from a combination of limited clinical experience and the perceived complexity of AI systems. Addressing these concerns early in medical education could help foster a more balanced perspective on the risks and benefits of AI in surgery. The identification of “lack of evidence of effectiveness” as a top barrier by both surgeons and surgical trainees underscores the need for robust clinical trials and real-world studies to demonstrate AI’s efficacy and safety in surgical applications. Our study also reveals concerns about the ethical and legal implications of AI in surgery, echoing challenges identified by Gerke et al. [17]. The concerns of medical students regarding malpractice highlight the need for clear legal frameworks as AI adoption increases. Interestingly, we observed a generational divide in attitudes towards AI transparency. Surgical trainees showed greater concern about this issue compared to established surgeons, possibly reflecting younger generations’ heightened demand for technological accountability. This disparity in perspectives across career stages emphasizes the importance of comprehensive approaches to AI integration.

This study of course has some limitations, the primary one being the uneven distribution of participants across different groups, with a significantly larger proportion of medical students compared to surgical trainees and consultant surgeons. This imbalance may skew the overall results and limit the generalisability of findings, particularly for the surgeon and trainee groups. Our study also lacks gender data, preventing consideration of potential gender-specific perspectives on AI in surgery. We did not employ technical measures to prevent multiple respondent submissions. However, our targeted distribution within professional surgical networks and the use of clear instructions requesting single participation were intended to minimize this risk. Additional limitations include the focus on the Irish healthcare context and the concentration on general surgery, which may limit the applicability of findings to other regions or surgical specialties. The voluntary nature of participation may have introduced self-selection bias, and the online survey format could have led to misunderstandings or technical issues affecting responses. Future research should address these limitations by ensuring a more balanced distribution of participants across career stages, including gender data and analysis, expanding the geographical scope and range of surgical specialties. A systematic review of survey studies from various countries could provide a more comprehensive comparison of global perspectives on AI in surgery.

## Conclusion

This study offers a nuanced view of AI perceptions in surgery, revealing both enthusiasm and caution across different experience levels in Ireland, highlighting the issues considered here and contributing to a more comprehensive understanding of AI perception in healthcare globally. It highlights a need for targeted education to address the gap between perceived and actual AI capabilities in surgery.

## Supplementary Information

Below is the link to the electronic supplementary material.

MOESM 1 (DOCX 2.03 MB)

## Data Availability

Data are available from the corresponding author on request.
