# Supplementary material for: Comparative analysis of perceptions on artificial intelligence in surgery: a survey study among surgeons and medical students in Ireland
Source: Ir J Med Sci. 2025 Sep 18;194(6):2341–9. doi: 10.1007/s11845-025-04079-z (PMC12769670; doi:10.1007/s11845-025-04079-z)
Supplement: Supplementary file 1 — (DOCX 2.03 MB) [file 11845_2025_4079_MOESM1_ESM.docx]

**Supplementary Material**

**Survey Questions**

1. I consent and would like to continue with the survey:

- Yes
- No

**Demographic Information**

1. Role:

- Medical Student, Surgeon, Surgical Trainee, Other (Please Specify)

1. Age:

- < 25
- 25-35
- 36-45
- 46-55
- 56+

1. Years of Clinical Experience (for Surgeons and Surgical Trainees only):

- 1-5 years
- 6-10 years
- 11-20 years
- 21+ years

1. Specialty (for Surgeons and Surgical Trainees only, Please Specify):

**Perceptions on AI's Potential in Surgery**

1. How familiar are you with the application of AI in surgical procedures?

- Very familiar, Somewhat familiar, Neutral, Not very familiar, Not at all familiar

1. Do you believe AI can enhance real-time decision-making during surgeries?

- Strongly agree, Agree, Neutral, Disagree, Strongly disagree

1. To what extent do you trust AI to provide real-time intraoperative guidance?

- Fully trust, Somewhat trust, Neutral, Somewhat distrust, Do not trust at all

1. How do you view the potential of AI in improving surgical outcomes?

- Very positive, Somewhat positive, Neutral, Somewhat negative, Very negative

1. Which aspects of surgical procedures do you believe would benefit most from the integration of AI?

- Preoperative planning and simulation
- Real-time decision-making during surgery
- Instrument precision and guidance
- Postoperative monitoring and predictions
- Patient data analysis and outcome predictions
- Other (Please Specify):

1. How would you describe the current status of AI in surgery?

- Always used, Often used, Sometimes used, Rarely used, Never used

**Training and Exposure**

1. Have you received any training or exposure related to AI in surgery?

- Yes
- No

1. If you answered Yes, how would you rate the quality of this training or exposure?

- Excellent, Very Good, Good, Fair, Poor

1. Would you be interested in more AI-focused training in the context of surgery?

- Extremely interested, Very interested, Neutral, Somewhat interested, Not interested

1. Do you think the current surgical curriculum should include more content about AI and its applications?

- Strongly agree, Agree, Neutral, Disagree, Strongly disagree

1. How do you believe AI can most effectively contribute to surgical training and education?

- Leveraging AI to offer personalised learning experiences, including adaptive learning paths that adjust to an individual's strengths.
- Utilising AI-driven simulations to provide realistic and diverse surgical scenarios for practice, thereby improving technical skills, decision-making, and readiness for real-life surgeries.
- Implementing AI systems that provide real-time feedback and detailed post- performance analysis to students, enabling them to identify areas for improvement and track their progress over time.
- Identifying areas for innovation in surgical techniques, thereby informing educational content and ensuring that trainees are exposed to the newest developments.
- Automating administrative tasks related to education management, such as scheduling, student assessments, and compliance tracking, to allow educators more time to focus on teaching and mentorship.
- Other (Please Specify):

**Concerns**

1. Which of the following do you see as the biggest risk in integrating AI into surgical procedures?

- Data privacy concerns
- Accuracy and reliability of AI predictions
- Integration with existing surgical tools and workflows Surgeon's dependence on AI over time
- Potential for technological malfunctions Liability and/or litigation concerns
- Other (Please Specify):

1. Which of the following do you perceive as the most significant barrier to adopting AI in the surgical setting?

- Lack of training
- Fear of malpractice
- Cost of implementation
- Lack of evidence of its effectiveness
- Other (Please Specify)

1. Do you believe AI can overshadow a surgeon's intuition or judgment?

- Strongly agree, Agree, Neutral, Disagree, Strongly disagree

1. Are you concerned about the transparency of AI algorithms in surgical applications?

- Not concerned, Slightly concerned, Neutral, Moderately concerned, Extremely concerned

**Liability and Ethics**

1. How concerned are you about potential liability issues when integrating AI into surgical procedures?

- Not concerned, Slightly concerned, Neutral, Moderately concerned, Extremely concerned

1. In the event of complications arising from AI integration in surgical procedures, who should bear the primary responsibility?

- The surgeon, The hospital, The AI manufacturer, A shared responsibility among multiple parties, Other (Please Specify):

**Final Thoughts**

Are there any other thoughts, comments, or insights you would like to share about AI as a decision-support tool in the context of surgery?

- Open-ended response
